# Prevalence, Mortality, Antifungal Resistance, and Risk Factors of Candidemia Among Cancer Patients in a Single Center of Southern China: A 10-Year Retrospective Study

**DOI:** 10.1155/jotm/2653325

**Published:** 2025-06-05

**Authors:** Limei Chen, Jieyu Li, Jianzhong Xie, Yansong Chen, Xiaolong Yu, Na Xin, Yanping Xiao, Guangjian Su, Zhenzhou Xiao

**Affiliations:** ^1^Department of Clinical Laboratory, Laboratory of Biochemistry and Molecular Biology Research, Fujian Cancer Hospital, Clinical Oncology School of Fujian Medical University, Fuzhou 350014, Fujian, China; ^2^Laboratory of Immuno-Oncology, Fujian Cancer Hospital, Clinical Oncology School of Fujian Medical University, Fuzhou 350014, Fujian, China; ^3^Department of Hospital Infection Management, Fujian Cancer Hospital, Clinical Oncology School of Fujian Medical University, Fuzhou 350014, Fujian, China

**Keywords:** antifungal resistance, bloodstream infection, cancer, *Candida*, candidemia, catheter-related bloodstream infection, risk factor, southern China

## Abstract

Cancer patients are at a high risk of *Candida* infections, and candidemia may aggravate the prognosis among patients with cancers. To investigate the incidence, mortality, risk factors, and antifungal resistance of candidemia among cancer patients, 100 inpatients with malignant solid tumors and candidemia in Fujian Province, southern China, during the period from January 2014 through December 2023 were recruited. Among the study subjects, *Candida albicans* was the predominant *Candida* species (50%), and the prevalence of candidemia showed an overall tendency towards a slight decline during the study period. *Candida tropicalis* showed 10.53% prevalence of resistance to fluconazole, voriconazole and itraconazole, while *C. albicans*, *Candida glabrata* and *Candida parapsilosis* were all totally susceptible to fluconazole, voriconazole, itraconazole and amphotericin B. The overall 30-day crude mortality of candidemia was 67% among cancer patients, and there was no significant difference between the mortality due to *Candida* catheter-related bloodstream infection (CRBSI) and bloodstream infection (BSI) (*p* = 0.59). Multivariate Cox regression analysis identified that the presence of cardiovascular diseases and use of two to three catheters (OR = 385.064, *p* = 0.005) increased the risk of candidemia among cancer patients. Our data demonstrate an overall tendency towards a slight decline in the prevalence of candidemia and a high mortality rate of candidemia among cancer patients in southeastern China from 2014 to 2023, and development of cardiovascular diseases and use of two to three catheters may increase the risk of candidemia among cancer patients.

## 1. Introduction

*Candida* is a genus of fungi that exists in human skin, mouth, vagina, and gastrointestinal tract [1]. Approximately 200 species of *Candida* have been identified until now; however, only a few species are human opportunistic and cause infections among debilitated or immunocompromised hosts [1]. The incidence of *Candida* bloodstream infections (BSI) has become 3.88 to 122/10^5^ among general population since the 21st century [1]. *Candida* has been recognized as the fourth most common cause of nosocomial BSI in the United States and the seventh in Europe [2, 3]. In addition, *Candida* is one of the most common invasive fungal pathogens among immunocompromised cancer patients, with an incidence rate of 0.15% to 1.5% [4].

Since candidemia has few specific clinical manifestations, its clinical diagnosis and treatment are difficult [5]. To date, the diagnosis of *Candida* BSI mainly depends on biomarkers and blood cultures [6]. Previous studies have shown that the biomarkers galactomannan and 1,3-β-d-glucan testing has shown comparable diagnostic sensitivity and specificity for *Candida* BSI, with 60% to 80% sensitivity and 90% specificity reported [7]. Currently, positive blood cultures remain the gold standard for diagnosis of candidemia; however, 1 to 3 days are required to observe positive growth of *Candida*, and the resultant delay in treatment is a well-known risk factor of mortality [5]. It has been demonstrated that the 30-day crude mortality of *Candida* BSI is 40% to 55% [8].

Cancer patients are immunocompromised due to long-term radiotherapy and/or chemotherapy, invasive procedures, and use of immunosuppressant and antimicrobial agents [9]. It has been shown that cancer patients are at a high risk of *Candida* infections and candidemia may aggravate the prognosis among patients with cancers [10]. Timely and rational use of antifungal agents is, therefore, of great importance among cancer patients complicated by candidemia [10]. Nevertheless, long duration for diagnosis of candidemia and missing diagnosis caused by false negatives urge the diagnosis of candidemia among cancer patients based on clinical manifestations and potential risk factors [11]. Therefore, identification of risk factors for host *Candida* BSI is required prior to development of antifungal treatment schemes [12].

It has been found that the distribution of common *Candida* species varies in countries, regions, institutions, and among patients with underlying diseases and use of antimicrobial agents [13]. Understanding the changes in the epidemiology and antifungal resistance of candidemia based on population- or institution-based surveillance may facilitate the improvements in the diagnosis and treatment of candidemia. However, there is little knowledge on the epidemiology and risk factors of candidemia among cancer patients in Fujian Province, China. This hospital-based study was therefore designed, with aims to investigate the incidence, mortality, risk factors, and antifungal resistance of candidemia among cancer patients in a single center of southeastern China during the 10 years from 2014 to 2023, so as to provide insights into improvements in prognosis, reduction in disease and economic burden, and improvements in quality of life among cancer patients with candidemia.

## 2. Methods

### 2.1. Subjects

During the period from January 2014 through December 2023, a total of 764,382 cancer patients were admitted to Fujian Cancer Hospital (Fuzhou, China), and solid cancer inpatients detected with at least once blood culture for *Candida* were enrolled in this study, while cancer patients with onset of candidemia within 30 days of the first onset or incomplete clinical data were excluded from the study. All patients with any onset of candidemia after 30 days of the first onset of candidemia were defined as incident cases. Subjects' demographics and clinical characteristics were collected from patients' medical records, including age, gender, invasive interventions, surgery, underlying diseases, *Candida* isolates, antifungal susceptibility, duration of hospital stay, and outcomes.

### 2.2. Case Definition

In this study, death from candidemia was defined as death within 30 days following presence of *Candida* in the first blood culture, without other causes of death, or with candidemia as the cause of death in death certificates, and survival from candidemia was defined as survival within 30 days following presence of *Candida* in blood cultures and without candidemia-related clinical symptoms. Candidemia was defined as disseminated infection caused by *Candida* in the blood, while catheter-related bloodstream infection (CRBSI) was defined as presence of candidemia among inpatients with intravascular catheters or within 48 h following removal of intravascular catheters, presenting fever (temperature of > 38°C), chills, or hypotension, and without other known sources of infection except intravascular catheters, and microbiological examinations detected the same *Candida* species and the same antimicrobial susceptibility tests from peripheral venous blood samples with those from catheter- or catheter-tip cultures (15 *Candida* colonies and more) [14].

### 2.3. Species Identification and Antifungal Susceptibility Test

Blood samples were tested daily for microbial growth using the BACTEC FX system (Becton Dickinson Diagnostic Instrument Systems; Sparks, MD, USA), and the microbial species were identified with the BD Phoenix 100 system (Becton Dickinson Diagnostic Instrument Systems). The susceptibility of fungal isolates to fluconazole, itraconazole, voriconazole, and amphotericin B was determined using the ATB FUNGUS 3 system (bioMérieux; Marcy, France) following the manufacturer's instructions, and the in vitro susceptibility of *Candida albicans*, *Candida glabrata*, *Candida parapsilosis*, and *Candida tropicalis* to fluconazole, itraconazole, voriconazole, and amphotericin B was interpreted using the clinical breakpoints (CBPs) defined by the latest version of the Clinical Laboratory Standards Institute (CLSI) [15] or European Committee on Antimicrobial Susceptibility Testing (EUCAST) guidelines during the study period from 2014 to 2023 [16]. Since the minimum inhibitory concentration (MIC) of CBPs for 5-fluorocytosine by CLSI or EUCAST were not available, this was not reported in the current study.

### 2.4. Ethical Statement

This study was approved by the Ethical Review Committee of Fujian Cancer Hospital (approval number: K2024-226-01). Written informed consent was obtained from all participants following a detailed description of the purpose of the study. All experimental procedures were performed in accordance with the Declaration of Helsinki and the Guidelines for Ethics Review of Life Sciences and Biomedical Studies Involving Humans.

### 2.5. Statistical Analysis

All data were entered into Microsoft Excel 2016 (Microsoft Corporation; Redmond, WA, USA), and all statistical analyses were performed using the statistical software SPSS version 26.0 (SPSS, Inc.; Chicago, IL, USA). All measurement data were tested for normality with Kolmogorov–Smirnov test. Normally distributed measure data were expressed as mean ± standard deviation (SD), and differences of means between groups were compared using Student *t* test, while non-normally distributed measure data were expressed as median (interquartile range), and comparisons of medians between groups were done with Mann–Whitney *U* test. Count data were described as numbers or proportions, and differences of means were tested for statistical significance with chi-square test or Fisher's exact test. Pairwise tests were done with Bonferroni correction. The survival curve was plotted using Kaplan–Meier estimates. In addition, univariate analysis was performed with chi-square test, and parameters with a statistical significance in univariate analysis were enrolled in a multivariate Cox regression model. A *p* value of < 0.05 was considered statistically significant.

## 3. Results

### 3.1. Distribution of *Candida* Species Among Cancer Patients

**Figure 1:**
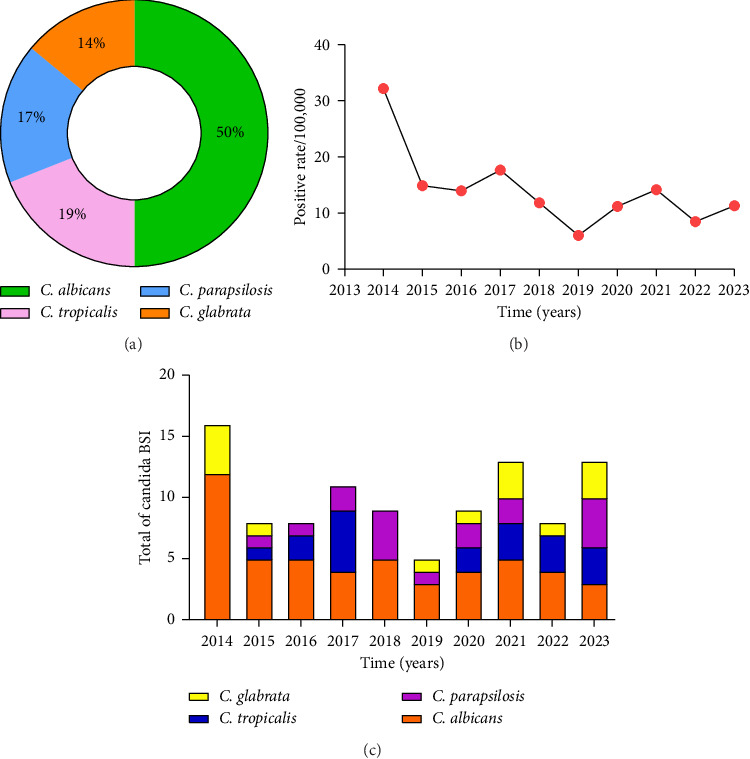
*Candida* species yielded from the blood cultures of cancer inpatients. (a) Percentage of *Candida* isolates; (b) annual prevalence of *Candida* isolates from 2014 to 2023; (c) number of isolates of different *Candida* species.

A total of 764,382 cancer patients were admitted to Fujian Cancer Hospital during the period from January 2014 to December 2023, and there were 100 inpatients detected with candidemia, including 50 cases with *C. albicans* infection, 19 cases with *C. tropicalis* infection, 17 cases with *C. parapsilosis* infection, and 14 cases with *C. glabrata* infection (Figure 1(a)). The prevalence of candidemia showed an overall tendency towards a slight decline (Figure 1(b)), and the prevalence of non-*Candida* infections showed a tendency towards a rise during the 10-year period from 2014 to 2023 (Figure 1(c)). However, *C. krusei* was not detected in blood specimens.

### 3.2. Susceptibility of *Candida* Isolates to Antifungal Agents

Antimicrobial susceptibility testing revealed that *C. tropicalis* showed 10.53% prevalence of resistance to fluconazole, voriconazole, and itraconazole, while *C. albicans*, *C. glabrata*, and *C. parapsilosis* were all totally susceptible to fluconazole, voriconazole, itraconazole, and amphotericin B (Table 1).

### 3.3. Mortality of Candidemia Among Cancer Patients

**Figure 2:**
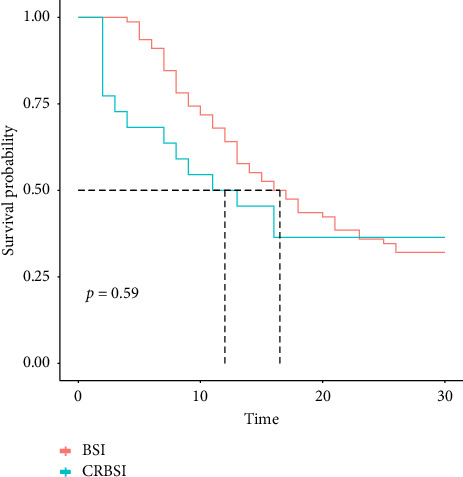
The 30-day crude mortality due to *Candida* catheter–related bloodstream infections and bloodstream infections.

The overall 30-day crude mortality of candidemia was 67% among cancer patients, and there was no significant difference between the mortality due to *Candida* CRBSI (72.73%, 14/22) and *Candida* BSI (65.38%, 53/78) (*p*=0.59) (Figure 2).

### 3.4. Risk Factors of Candidemia Among Cancer Patients

Univariate analysis showed that there were significant differences in the prevalence of candidemia among cancer patients in terms of presence of liver diseases, presence of more than two types of underlying diseases, radiotherapy/chemotherapy, surgery, use of antimicrobial agents, number of catheters used, duration of catheters use, mechanical ventilation, and tumor stage (Table 2). Multivariate Cox regression analysis identified that presence of cardiovascular disease (OR = 126.626, *p*=0.036) and use of two to three catheters (OR = 385.064, *p*=0.005) increased the risk of candidemia among cancer patients (Table 3).

### 3.5. Comparison of Demographic and Clinical Characteristics of Cancer Patients With *Candida* CRBSI and BSI

Univariate analysis showed that stage IV cancer patients had a significantly higher prevalence rate of *Candida* BSI than that of *Candida* CRBSI, and a higher detection rate of *C. glabrata* was found among cancer patients with *Candida* CRBSI than those of *C. albicans*, *C. parapsilosis*, and *C. tropicalis* (Table 4).

## 4. Discussion

Cancer has been identified as a risk factor of candidemia, and cancer patients are at a high risk of candidemia [17]. According to the guidelines for the management of candidiasis in Europe and the United States, critically ill patients with risk factors of invasive candidiasis and without other known causes of fever are recommended to be given empirical antifungal therapy [18, 19]. In the current study, *C. albicans* was found to be the predominant pathogen of candidemia in Fujian Province from 2014 to 2023; however, the prevalence of non-*Candida* infections showed a tendency towards a rise during the 10-year period, which was in agreement with previous reports [20, 21].

Currently, the Candida species consisted of 50% of *C. albicans* and 50% of non-*C. albicans* [1]. Nevertheless, the distribution of *Candida* species causing candidemia has recently shifted from *C. albicans* to non-*C. albicans* across the world [13], which was consistent with the findings from this study. In the present study, *C. tropicalis* was the dominant non-*C. albicans* species, which was similar to the findings in Columbia [22], and in different regions of China [23–25], and was not consistent with the report by Otto and colleagues [26]. *C. tropicalis* and *C. parapsilosis* have been identified as the two most common *Candida* species among malignant solid tumor patients [27, 28]. These findings indicate that the distribution of *Candida* species varies in regions, institutions, and study populations [13]. Previous studies have identified the use of three or more broad-spectrum antimicrobials and immunosuppression as risk factors of *Candida famata* and *Candida pelliculosa* candidemia among neonates and adults [29–31]; however, this was not found in the current study. Further studies to identify the risk factors for *C. famata* and *C. pelliculosa* candidemia among cancer patients are encouraged.

In this study, antifungal susceptibility tests revealed an overall high susceptibility of *Candida* isolates to antifungal agents fluconazole, voriconazole, itraconazole, and amphotericin B, and *C. albicans*, *C. glabrata* and *C. parapsilosis* isolates were all totally susceptible to fluconazole, voriconazole, itraconazole, and amphotericin B, which was in agreement with previous reports [32]. Our antifungal susceptibility testing showed an 89.47% susceptibility to fluconazole in *C. tropicalis* isolates, and the prevalence of susceptibility to fluconazole was 98.9% in *C. tropicalis* isolates, from Latin America and 94.9% from North America [32]. In addition, our study showed that the prevalence of voriconazole-susceptible *C. tropicalis* isolates was 89.47%, which was 86% in the Asia-Pacific region, China, Australia, and Europe [33–36]. Currently, fluconazole is the most common antifungal agent, followed by echinocandin and voriconazole [37]. Fluconazole has shown higher safety and tolerance, and lower cost than amphotericin B, which has been given higher attention in clinical practices, although amphotericin B has a high in vitro antifungal activity against *Candida* species [38]. The United States and European guidelines for management of candidiasis have recommended echinocandin as empirical therapy among critically ill patients or patients with fluconazole-resistant candidemia [18, 19]; however, the susceptibility to echinocandin was not tested in *Candida* isolates in this study. Further studies to test the susceptibility to echinocandin in *Candida* isolates from cancer patients seem justified.

Although the types of antifungal drugs have recently increased [37], the overall 30-day crude mortality of candidemia is high among cancer patients and the high mortality may be attributed to immunodeficiency and delay in use of antifungal drugs among cancer patients [10]. It is widely known that the mortality of candidemia is attributed to failure in host defense mechanisms, patients' potential diseases and complications, relative virulence of *Candida* isolates, improper treatment, or delay in treatment [39]. Even if all antifungal therapy is administered through a central line catheter, no delay will happen [40]. In this study, the overall 30-day crude mortality of candidemia was 67% among cancer patients, which was higher than the results reported by Otto and colleagues [26]. This may be associated with patients' immunodeficiency, long-term radiotherapy/chemotherapy, use of immunosuppressant, and invasive procedures. The virulence of *C. parapsilosis* and *C. krusei* is reported to be lower than that of *C. albicans*, *C. tropicalis*, and *C. glabrata* in animal models [41]. In this study, relatively highly virulent *C. albicans* and *C. tropicalis* were dominant that caused candidemia among cancer patients, which may be associated with the high mortality. Our data showed no significant difference between the mortality due to *Candida* CRBSI and *Candida* BSI (72.73% vs. 65.38%, *p*=0.59), which was in agreement with previous reports showing the 46% to 75% mortality of *Candida* CRBSI [42, 43]. The difference in mortality of candidemia may be attributed to study design, study subjects, treatment decision, and sample size. Previous studies have shown a relatively lower mortality of *Candida* CRBSI among noncancer patients [44]. Since the mortality of candidemia is high among cancer patients [10], preventive and empirical antifungal therapy is strongly recommended for patients with severe disease [29, 30]. Because of individual potential risk factors, the severe liver and kidney toxicity of antifungal agents may aggravate cancer patients' conditions [45], when inappropriate prevention may facilitate non-*C. albicans* growth, resulting in a high difficulty in the determination of the timing for preventive antifungal therapy. In this study, we identified the risk factors of candidemia among cancer patients, and a case-control study was employed, so as to provide insights into antifungal therapy of candidemia among cancer patients.

In this study, univariate analysis revealed that number of catheters used, duration of catheters use, mechanical ventilation, short-term use of antimicrobial agents, surgery, and radiotherapy/chemotherapy were associated with an elevated risk of candidemia, which was consistent with previous reports [46, 47], and stage IV cancer patients has a higher risk of candidemia than stage I to III patients, which may be associated with patients' immunodeficiency, long-term radiotherapy/chemotherapy, and use of immunosuppressant. Increasing evidence has shown that cancer is an independent risk factor of candidemia [10, 11, 26]; however, there is no knowledge on the cancer stage prevalence of candidemia. In this study, we found a higher prevalence rate of candidemia among malignant solid tumor patients with underlying liver diseases than those without underlying liver diseases. Previous studies have identified use of catheters as an independent risk factor of candidemia [48], which was in agreement with the results from our multivariate Cox regression analysis. In addition, we found that the underlying cardiovascular diseases increased the risk of candidemia, which may be attributed to geographical factors and individuals with potential diseases. Further studies to examine the contribution of cardiovascular diseases to candidemia are required. In this study, we found no significant differences in the prevalence of candidemia between cancer patients in terms of parenteral nutrition or recent surgery, which is not in agreement with previous reports [49, 50]. This may be attributed to only 11 cases with parenteral nutrition in our center. In addition, most cases were at an advanced stage in our center, which may have a higher likelihood of developing candidemia. In the present study, univariate analysis showed that stage IV cancer patients had a significantly higher prevalence rate of *Candida* BSI than that of CRBSI, and a higher detection rate of *C. glabrata* was found among cancer patients with *Candida* CRBSI than those of *C. albicans*, *C. parapsilosis*, and *C. tropicalis*. In addition, mortality was comparable between those with *Candida* CRBSI and *Candida* BSI (72.73% vs. 65.38%, *p*=0.59). Previous studies have demonstrated that the catheter-related mortality is 46% to 75% [51]; therefore, early removal of catheters may improve the prognosis among patients with candidemia [19].

Recently, the prevalence of candidemia has shown a tendency towards a slight decline among cancer patients in southeastern China from 2014 to 2023, which may be attributed to COVID-19 pandemic. However, an increase has been observed in candidemia patients during the COVID-19 pandemic [52]. Active infection control interventions and empiric antifungal treatment are effective to reduce the mortality of candidemia among cancer patients.

In summary, the results of the present study demonstrate a high mortality rate of candidemia among malignant solid tumor patients, and the prevalence of non-*C. albicans* infections showed a tendency towards a rise in Fujian Province, southeastern China, from 2014 to 2023. Our single-center analysis shows that blood-derived *Candida* isolates are highly susceptible to currently common antifungal drugs, and stage IV cancers are an independent risk factor of candidemia among malignant solid tumor patients. Our findings facilitate the diagnosis and treatment of candidemia and the improvements in prognosis among cancer patients.

## Figures and Tables

**Table 1:** In vitro antifungal susceptibility test of *Candida* isolates.

Species (*n* = 100)	Antifungal agent	MIC (μg/mL)			No. (%) of isolates by new CBPs		
		Ranges	MIC_50_	MIC_90_	S	SDD	R
*C. albicans* (*n* = 50)	Fluconazole	1–128	≤ 1	1	50 (100%)	0	0
	Voriconazole	0.06–8	≤ 0.06	0.06	50 (100%)	0	0
	Itraconazole	0.125–4	≤ 0.125	0.125	50 (100%)	0	0
	Amphotericin B	0.5–16	≤ 0.5	0.5	50 (100%)	0	0

*C. glabrata* (*n* = 14)	Fluconazole	1–128	≤ 1	1	14 (100%)	0	0
	Voriconazole	0.06–8	≤ 0.06	0.06	14 (100%)	0	0
	Itraconazole	0.125–4	0.125	0.25	ND	ND	ND
	Amphotericin B	0.5–16	≤ 0.5	0.5	14 (100%)	0	0

*C. parapsilosis* (*n* = 17)	Fluconazole	1–128	≤ 1	1	17 (100%)	0	0
	Voriconazole	0.06–8	≤ 0.06	0.06	17 (100%)	0	0
	Itraconazole	0.125–4	≤ 0.125	0.125	17 (100%)	0	0
	Amphotericin B	0.5–16	≤ 0.5	0.5	17 (100%)	0	0

*C. tropicalis* (*n* = 19)	Fluconazole	1–128	1	8	17 (89.47%)	0	2 (10.53%)
	Voriconazole	0.06–8	0.06	1	17 (89.47%)	0	2 (10.53%)
	Itraconazole	0.125–4	0.25	1	17 (89.47%)	0	2 (10.53%)
	Amphotericin B	0.5–16	≤ 0.5	0.5	19 (100%)	0	0

*Note:* S, susceptible; R, resistant. CBPs for *Candida* susceptibility to fluconazole and voriconazole are obtained from CLSI [15], while CBPs for susceptibility of *Candida* against itraconazole and amphotericin B are from EUCAST [16].

Abbreviations: MIC, minimum inhibitory concentration; ND, not detected; SDD, susceptible-dose dependent.

**Table 2:** Univariate analysis of risk factors of candidemia among cancer patients.

Independent variable		Total subjects (*n* = 200)	Candidemia		*χ* ^2^ */t/Z* value	*p* value
			Uninfected (*n* = 100)	Infected (*n* = 100)		
Department	ICU	58	30 (51.7)	28 (48.3)	—	1.000^∗^
	Neck surgery	4	2 (50.0)	2 (50.0)		
	Gynecology	20	10 (50.0)	10 (50.0)		
	Gastroenterology	42	21 (50.0)	21 (50.0)		
	Digestive surgery	54	26 (48.1)	28 (51.9)		
	Respiratory medicine	2	1 (50.0)	1 (50.0)		
	Lymphoma internal medicine	10	5 (50.0)	5 (50.0)		
	Thoracic surgery	10	5 (50.0)	5 (50.0)		

Gender	Men	110	54 (49.1)	56 (50.9)	0.081	0.776
	Women	90	46 (50.9)	44 (48.9)		

Age (years)		200	58.5 ± 11.8	58.1 ± 12.7	−0.237	0.813

Diagnosis	Intestinal, hepatic, biliary, and pancreatic cancers	84	42 (50.0)	42 (50.0)	—	1.000^∗^
	Lung cancer	12	6 (50.0)	6 (50.0)		
	Gynecologic cancer	28	14 (50.0)	14 (50.0)		
	Lymphoma	10	5 (50.0)	5 (50.0)		
	Esophageal and gastric cancers	62	31 (50.0)	31 (50.0)		
	Other cancers	4	2 (50.0)	2 (50.0)		

Stage	I	9	7 (77.8)	2 (22.2)	—	< 0.001^∗^
	II	20	16 (80.0)	4 (20.0)b		
	III	35	29 (82.9)	6 (17.1)b		
	IV	136	48 (35.3)	88 (64.7)a		

Underlying disease	None	128	79 (61.7)	49 (38.3)	39.937	< 0.001
	Cardiovascular diseases	20	9 (45.0)	11 (55.0)		
	Diabetes	17	11 (64.7)	6 (35.3)		
	Liver disease	18	0 (0.0)	18 (100.0)		
	Presence of 2 and more above diseases	17	1 (5.9)	16 (94.1)		

Radiotherapy/chemotherapy	No	68	52 (76.5)	16 (23.5)	28.877	< 0.001
	Yes	132	48 (36.4)	84 (63.6)		

Surgery	No	130	78 (60.0)	52 (40.0)	14.857	< 0.001
	Yes	70	22 (31.4)	48 (68.6)		

Use of antibiotics	No	117	93 (79.5)	24 (20.5)	98.054	< 0.001
	Yes	83	7 (8.4)	76 (91.6)		

No. catheters used	0-1	99	71 (71.7)	28 (28.3)	36.984	< 0.001^∗^
	2-3	101	29 (28.7)	72 (71.3)		

Duration of catheterization (day)		200	10 (6, 21)	23 (10, 39.75)	−5.535	< 0.001

Inpatient in ICU	No	132	70 (53.0)	62 (47.0)	1.426	0.232
	Yes	68	30 (44.1)	38 (55.9)		

Mechanical ventilation	No	168	97 (57.7)	71 (42.3)	25.149	< 0.001
	Yes	32	3 (9.4)	29 (90.6)		

Parenteral nutrition	No	189	96 (50.8)	93 (49.2)	0.866	0.352
	Yes	11	4 (36.4)	7 (63.6)		

^∗^Fisher's exact test.

**Table 3:** Multivariate Cox regression analysis of risk factors of candidemia among cancer patients.

Independent variable		*β*	SE	Wald *χ*^2^	*P*	HR	95% CI	
							Lower limit	Upper limit
Cancer stage	II	−0.525	0.913	0.330	0.566	0.592	0.099	3.545
	III	−0.284	0.863	0.108	0.743	0.753	0.139	4.089
	IV	−0.729	0.797	0.837	0.360	0.482	0.101	2.301

Underlying disease	Cardiovascular diseases	−0.142	0.380	0.140	0.708	0.867	0.412	1.827
	Diabetes	−0.009	0.446	0.000	0.984	0.991	0.413	2.378
	Liver disease	−0.624	0.303	4.246	0.039	0.536	0.296	0.970
	Presence of 2 and more above diseases	−0.137	0.316	0.187	0.666	0.872	0.469	1.621

Radiotherapy/chemotherapy	Yes	0.164	0.376	0.191	0.662	1.179	0.564	2.465

Surgery	Yes	−0.630	0.248	6.447	0.011	0.533	0.328	0.866

Use of antibiotics	Yes	0.074	0.245	0.090	0.764	1.077	0.665	1.742

No. catheters used	2–3	−0.191	0.268	0.508	0.476	0.826	0.488	1.397

Duration of catheterization (day)		−0.007	0.005	1.902	0.168	0.993	0.984	1.003

Mechanical ventilation	Yes	0.526	0.262	4.048	0.044	1.693	1.014	2.827

**Table 4:** Comparison of demographic and clinical characteristics of cancer patients with *Candida* catheter–related bloodstream infections and bloodstream infections.

Characteristic		Total subjects (*n* = 100)	Type of infection		*χ* ^2^ */t* value	*p* value
			Bloodstream infection (*n* = 78)	Catheter-related bloodstream infection (*n* = 22)		
Department	ICU	28	21 (75.0)	7 (25.0)	—	0.846^∗^
	Neck surgery	2	2 (100.0)	0 (0.0)		
	Gynecology	10	6 (60.0)	4 (40.0)		
	Gastroenterology	21	18 (85.7)	3 (14.3)		
	Digestive surgery	28	22 (78.6)	6 (21.4)		
	Respiratory medicine	1	1 (100.0)	0 (0.0)		
	Lymphoma internal medicine	5	4 (80.0)	1 (20.0)		
	Thoracic surgery	5	4 (80.0)	1 (20.0)		

Gender	Men	56	44 (78.6)	12 (21.4)	0.024	0.876
	Women	44	34 (77.3)	10 (22.7)		

Age (years)		100	58.3 ± 12.9	57.1 ± 12.2	0.384	0.701

Diagnosis	Intestinal, hepatic, biliary, and pancreatic cancers	42	33 (78.6)	9 (21.4)	—	0.672^∗^
	Lung cancer	6	6 (100.0)	0 (0.0)		
	Gynecologic cancer	14	9 (64.3)	5 (35.7)		
	Lymphoma	5	4 (80.0)	1 (20.0)		
	Esophageal and gastric cancers	31	24 (77.4)	7 (22.6)		
	Other cancers	2	2 (100.0)	0 (0.0)		

Cancer stage	I	2	1 (50.0)a	1 (50.0)	—	0.044^∗^
	II	4	2 (50.0)a	2 (50.0)		
	III	6	3 (50.0)a	3 (50.0)		
	IV	88	72 (81.8)b	16 (18.2)		

Underlying disease	None	49	39 (79.6)	10 (20.4)	—	0.954
	Cardiovascular disease	11	9 (81.8)	2 (18.2)		
	Diabetes	6	5 (83.3)	1 (16.7)		
	Liver disease	18	13 (72.2)	5 (27.8)		

Radiotherapy/chemotherapy	No	16	14 (87.5)	2 (12.5)	—	0.512
	Yes	84	64 (76.2)	20 (23.8)		

Surgery	No	52	41 (78.8)	11 (21.2)	0.045	0.832
	Yes	48	37 (77.1)	11 (22.9)		

Use of antibiotics	No	24	18 (75.0)	6 (25.0)	0.166	0.684
	Yes	76	60 (78.9)	16 (21.1)		

No. catheters used	0-1	28	22 (78.6)	6 (21.4)	0.007	0.931
	2-3	72	56 (77.8)	16 (22.2)		

Duration of catheterization (day)		100	23 (10.39)	28 (9, 70.25)	−0.695	0.487

Inpatient in ICU	No	62	49 (79.0)	13 (21.0)	0.101	0.750
	Yes	38	29 (76.3)	9 (23.7)		

Mechanical ventilation	No	71	56 (78.9)	15 (21.1)	0.109	0.742
	Yes	29	22 (75.9)	7 (24.1)		

Parenteral nutrition	No	93	71 (76.3)	22 (23.7)	—	0.342
	Yes	7	7 (100.0)	0 (0.0)		

*Candida* species	*C. albicans*	50	36 (72.0)	14 (28.0)	—	0.001^∗^
	*C. glabrata*	14	7 (50.0)	7 (50.0)		
	*C. parapsilosis*	17	17 (100.0)	0 (0.0)		
	*C. tropicalis*	19	42 (84.0)	1 (5.3)		

^∗^Fisher's exact test.

## Data Availability

The data that support the findings of this study are available from the corresponding author upon reasonable request.
